# Adverse Gastrointestinal Events With Sodium Polystyrene Sulfonate Use in Patients on Maintenance Hemodialysis: An International Cohort Study

**DOI:** 10.1177/20543581231172405

**Published:** 2023-06-21

**Authors:** Ana Cecilia Farfan Ruiz, Ranjeeta Malick, Emily Rhodes, Edward Clark, Greg Hundemer, Angelo Karaboyas, Bruce Robinson, Roberto Pecoits, Manish M. Sood

**Affiliations:** 1 Division of Nephrology, Department of Medicine, Ottawa Hospital Research Institute, The Ottawa Hospital, University of Ottawa, ON, Canada; 2 Institute for Clinical Evaluative Sciences, Ottawa, ON, Canada; 3 Arbor Research Collaborative for Health, MI, USA

**Keywords:** SPS, hyperkalemia, hemodialysis, DOPPS, international, gastrointestinal events

## Abstract

**Background:**

There are concerns regarding the gastrointestinal (GI) safety of sodium polystyrene sulfonate (SPS), a medication commonly used in the management of hyperkalemia.

**Objective:**

To compare the risk of GI adverse events among users versus non-users of SPS in patients on maintenance hemodialysis.

**Design:**

International prospective cohort study.

**Setting:**

Seventeen countries (Dialysis Outcomes and Practice Patterns Study [DOPPS] phase 2-6 from 2002 to 2018).

**Patients:**

50 147 adults on maintenance hemodialysis.

**Measurements:**

An adverse GI event defined by a GI hospitalization or GI fatality with SPS prescription compared with no SPS prescription.

**Methods:**

Overlap propensity score–weighted Cox models.

**Results:**

Sodium polystyrene sulfonate prescription was present in 13.4% of patients and ranged from 0.42% (Turkey) to 20.6% (Sweden) with 12.5% use in Canada. A total of 935 (1.9%) adverse GI events (140 [2.1%] with SPS, 795 [1.9%] with no SPS; absolute risk difference 0.2%) occurred. The weighted hazard ratio (HR) of a GI event was not elevated with SPS use compared with non-use (HR = 0.93, 95% confidence interval = 0.83-1.6). The results were consistent when examining fatal GI events and/or GI hospitalization separately.

**Limitations:**

Sodium polystyrene sulfonate dose and duration were unknown.

**Conclusions:**

Sodium polystyrene sulfonate use in patients on hemodialysis was not associated with a higher risk of an adverse GI event. Our findings suggest that SPS use is safe in an international cohort of maintenance hemodialysis patients.

## Abrégé

**Contexte:**

Des préoccupations sont soulevées quant à l’innocuité gastro-intestinale (GI) du sulfonate de polystyrène sodique (SPS), un médicament couramment utilisé dans la gestion de l’hyperkaliémie.

**Objectif:**

Comparer dans une population de patients sous hémodialyse d’entretien le risque d’effets indésirables gastro-intestinaux chez les utilisateurs du SPS par rapport aux patients non-utilisateurs.

**Conception:**

Étude de cohorte prospective internationale.

**Cadre:**

17 pays (phases 2 à 6 de l’essai DOPPS [de 2002 à 2018]).

**Sujets:**

50 147 adultes sous hémodialyse d’entretien.

**Mesures:**

La comparaison entre les événements gastro-intestinaux indésirables, définis par une hospitalisation ou un décès en lien avec un problème gastro-intestinal, selon que les patients avaient ou non une prescription de SPS.

**Méthodologie:**

Modèles de Cox pondérés par le score de propension au chevauchement.

**Résultats:**

Dans l’ensemble de la cohorte, 13,4 % des patients avaient une prescription de SPS; l’usage de SPS variait selon les pays entre 0,42 % (Turquie) et 20,6 % (Suède) avec 12,5 % au Canada. En tout, 935 (1,9 %) événements GI indésirables sont survenus dans l’ensemble de la cohorte, soit 140 (2,1 %) chez les patients avec prescription de SPS et 795 (1,9 %) chez les patients sans prescription de SPS (différence de risque absolue: 0,2 %). Le rapport de risque (RR) pondéré d’un événement GI n’était pas plus élevé avec l’utilisation de SPS (RR = 0,93; IC 95 %: 0,83-1,6). Les résultats étaient cohérents lorsque l’on a examiné séparément les événements gastro-intestinaux (hospitalisation et/ou décès).

**Limites:**

La dose et la durée du traitement par SPS étaient inconnues.

**Conclusion:**

L’utilisation de SPS chez les patients sous hémodialyse n’a pas été associée à un risque plus élevé d’événements indésirables d’origine gastro-intestinale. Nos résultats suggèrent que l’utilisation du SPS est sans danger dans la cohorte internationale de patients sous hémodialyse d’entretien étudiée.

## Introduction

Individuals on maintenance dialysis are at an exceedingly high risk of recurrent hyperkalemia with regional differences in prevalence and severity.^1,2^ Of concern, step-wise elevations in potassium are associated with higher all-cause mortality and, as such, potassium levels are carefully monitored and often require additional therapies.^3,4^ Dietary potassium restriction, discontinuation of potassium-elevating medications, augmentation in urinary excretion (if applicable), and modifications in dialytic prescription represent viable means of obtaining potassium control.^5,6^

One of the most used therapies to reduce or mitigate hyperkalemia are gastrointestinal (GI) potassium binders. Sodium polystyrene sulfonate (SPS), first approved in by the US Food and Drug Administration (FDA) in June 1958, is the most frequently prescribed potassium cation exchange resin globally.^7^ Since introduction and widespread use, GI injury, mainly colonic necrosis, has repeatedly been attributed to the use of both oral and rectal SPS–sorbitol coadministration.^8^ A large body of basic science, translational, case reports, case series, and epidemiologic studies have linked SPS use with fatal and non-fatal GI injury, both to the upper and lower GI tract.^9-23^ The risk of GI injury appears high with lower kidney function, concurrent sorbitol administration, and prolonged GI luminal exposure due to constipation.^21^ Recently, a large retrospective population-based cohort study from our group including 40 040 older adults similarly documented a nearly 2-fold higher incidence of non-fatal hospitalization or emergency room visit for serious adverse GI events in 20 020 SPS users within 30 days of first prescription compared with 20 020 matched non-users.^20^

However, few studies to date have focused solely on patients requiring kidney replacement therapy, specifically maintenance hemodialysis. Hemodialysis patients are exposed, on average, to the highest mean serum potassium, are at high risk of recurrent hyperkalemia, and are commonly constipated placing them at high risk for GI injury.^20,22,24,25^ A recent French nationwide dialysis registry study reported no significant increase in emergency room visits or hospitalizations for adverse GI events in 43 771 SPS users.^26^ Whether this was region-specific or consistent in a larger, international cohort remains unclear. As such, we set out to examine the association of SPS use and adverse GI events among hemodialysis patients using the international prospective cohort study (Dialysis Outcomes and Practice Patterns Study, DOPPS). We hypothesized that SPS use was associated with an elevated risk of GI injury in patients on maintenance hemodialysis.

## Methods

### Study Design and Population

We conducted a retrospective cohort study of all adults (18 years or older) on hemodialysis captured in DOPPS. The DOPPS is an international dialysis cohort that collects information on patients on maintenance hemodialysis. Dialysis Outcomes and Practice Patterns Study uses a 2-stage random cluster sample where random faculties in a geographic region are selected followed by a random selection of patients within the facilities.^27^ Dialysis Outcomes and Practice Patterns Study collects detailed information on individual patients’ demographics, comorbid illnesses, medications, laboratory values, dialysis characteristics, hospitalizations, and mortality. Details on the DOPPS study design and methodology can be found elsewhere.^27^ We included all individuals enrolled in DOPPS phases 2 to 6 (2002-2018) with known vascular access and where cause of death data was collected. Individuals were censored at first occurrence of death, study outcome, loss of follow-up, or end of study period (see Supplementary Table 1 for study cohort creation). We excluded individuals with missing vascular access information (n = 1872) and a missing study end date (n = 7). Regional ethics board approval was obtained for this de-identified retrospective study form the Ottawa Hospital Regional Ethics Board (REB).

### Exposure

The study exposure was a first SPS prescribed (new user) on periodic (updated every 4 months) medication review since dialysis initiation. Information on dose of SPS use was not available.

### Study Outcome

The study outcome was an adverse GI event requiring a hospitalization or a GI fatality. Non-fatal GI events were defined as hospitalization for (1) gastritis/peptic ulcer disease, (2) gastric surgery/resection, or (3) colectomy/colon surgery. Fatal GI events were defined as death due to a direct GI primary cause as deemed by a physician. Fatal GI events were determined using cause of death data and were defined by death by (1) mesenteric infarction/ischemic bowel, (2) perforation of peptic ulcer, or (3) perforation of bowel. We further examined GI hospitalization or a GI fatality separately.

### Covariates

Demographics (age, sex, era, country), baseline comorbid illnesses (GI bleeding, hypertension, diabetes, stroke, peripheral vascular disease, congestive heart failure, coronary artery disease, cancer, lung disease, neurologic disorder, psychiatric disorder, deep vein thrombosis, parathyroidectomy), medications (diuretic [loop or thiazide], proton pump inhibitor, angiotensin-converting enzyme inhibitor, angiotensinogen receptor blocker, beta-blocker, acetylsalicylic acid [ASA]), pre-dialysis laboratory values (hemoglobin, phosphate, albumin, potassium), and dialysis characteristics (vintage, treatment time, dialysate potassium bath, potassium gradient defined as serum minus the dialysate potassium)^28,29^ were included. The potassium gradient was previously reported to be associated with adverse events in the dialysis population in a dose-dependent manner.^28^

### Statistical Analysis

We examined differences in baseline characteristics with and without SPS use by standardized differences. Standardized differences describe differences between group means relative to the pooled standard deviation, are less sensitive to large sample sizes than traditional hypothesis testing, and a significant difference is considered to be 10% or greater.^30^ We examined the association of SPS use versus non-use and the study outcomes using cause-specific Cox proportional hazards models.^31^ Schoenfeld residuals were examined to test the proportionality assumption. We examined SPS exposure as intention to treat and only the first GI hospitalization event was considered. We used overlap propensity score weighting to create balance between the analytical study groups (SPS vs no SPS).^32^ A propensity score for SPS exposure was calculated using all the baseline covariates listed in Table 1 and then overlap-weighted analyses were done. Overlap weights assign less weight to observations with extreme propensity scores and more weight to those with propensity scores close to 0.5.^33^ This method creates a balanced cohort in terms of the baseline variables and is less influenced by extreme outliers over-dominating often requiring truncation with inverse probability treatment weighting.^34^ Post-weighting balance was examined using standardized differences and models were additionally adjusted for variables with imbalance (SD > 0.1), specifically dialysis vintage and the potassium gradient. We conducted all analyses with SAS software, version 9.4 (SAS Institute Inc., Cary, North Carolina). Confidence intervals that did not overlap with 1 or *P* <.05 were treated as statistically significant.

**Table 1.** Baseline Characteristics for the Total Study Cohort and Stratified By Sodium Polystyrene Sulfonate (SPS) Exposure.

Characteristics	Total	SPS	No SPS	Before weighting	After weighting
	N (%)	N (%)	N (%)	Std diff.^a^	Std diff.^a^
N	50 147	6735 (13.4%)	43 412 (86.6%)		
Demographics					
Age (mean, SD)	64.0 (14.6)	63.3 (14.4)	64.1 (14.6)	0.05	<0.0001
Sex (male)	30 383 (60.6)	4086 (60.7)	26 297 (60.6)	0.002	<0.0001
Era (%)					
2002-2004	6252 (9.9)	588 (8.3)	5664 (10.1)	0.06	<0.0001
2005-2008	18 062 (28.5)	2240 (31.5)	15 822 (28.1)	0.07	<0.0001
2009-2011	15 418 (24.4)	1670 (23.5)	13 748 (24.5)	0.02	<0.0001
2012-2015	15 015 (23.7)	1437 (22.2)	13 578 (24.1)	0.09	<0.0001
2016-2018	8576 (13.5)	1172 (16.5)	7404 (13.2)	0.09	<0.0001
Comorbid illness					
GI bleeding	2487 (5.0)	243 (3.6)	2244 (5.2)	0.08	<0.0001
HTN	42 269 (84.3)	5732 (85.1)	36 537 (84.2)	0.03	<0.0001
Diabetes	20 628 (41.1)	2326 (34.5)	18 302 (42.2)	**0.16**	<0.0001
Stroke	7998 (16.0)	999 (14.8)	6999 (16.1)	0.04	<0.0001
PVD	13 016 (26.0)	1841 (27.3)	11 175 (25.7)	0.04	<0.0001
CHF	13 861 (27.6)	1814 (26.9)	12 047 (27.8)	0.02	<0.0001
CAD	20 567 (41.0)	2608 (38.7)	17 959 (41.4)	0.05	<0.0001
Cancer	7025 (14.0)	965 (14.3)	6060 (14.0)	0.01	<0.0001
Lung disease	5944 (11.8)	771 (11.4)	5173 (11.9)	0.01	<0.0001
Neurologic disorder	5320 (10.6)	670 (10.0)	4650 (10.7)	0.03	<0.001
Psychiatric disorder	7705 (15.4)	1027 (15.2)	6678 (15.4)	0.004	<0.0001
DVT	1877 (3.74)	292 (4.3)	1585 (3.65)	0.03	<0.0001
Parathyroidectomy	2581 (5.2)	489 (7.3)	2092 (4.8)	0.10	<0.0001
Medications					
Diuretic	16 318 (32.5)	2364 (35.1)	13 954 (32.1)	0.06	<0.0001
PPI	21 332 (42.5)	3029 (45.0)	18 303 (42.2)	0.06	<0.0001
ACEI	9614 (19.2)	1454 (21.6)	8160 (18.8)	0.07	<0.0001
ARB	10 672 (21.3)	1600 (23.8)	9072 (20.9)	0.07	<0.0001
Beta-blocker	20 355 (40.6)	2941 (43.7)	17 414 (40.1)	**0.07**	<0.0001
ASA	17 731 (35.4)	2422 (36.0)	15 309 (35.3)	0.01	<0.0001
Laboratory values					
Hemoglobin (mean, SD, g/L)	110.8 (15.3)	111.9 (14.8)	110.6 (15.3)	0.09	<0.0001
Phosphate (mean, SD, mg/dl)	5.2 (1.7)	5.5 (1.8)	5.2 (1.6)	**0.15**	<0.0001
Albumin (mean, SD, g/L)	36.6(5.2)	36.6 (5.0)	35.8 (5.5)	0.01	<0.0001
Potassium (mean, SD, mEq/L)	4.9 (0.8)	5.2 (0.8)	4.9 (0.8)	**0.42**	**0.60**
% <6 mEq/L(n = 37 945)	34 453 (90.8)	4422 (83.9)	30 031 (91.9)	**0.25**	**0.23**
Dialysis characteristics					
Vascular access type					
AVF	32 942 (65.7)	4734 (70.3)	28 208 (65.0)	0.11	<0.0001
AVG	4603 (9.2)	499 (7.4)	4104 (9.4)	0.07	<0.0001
Catheter	12 442 (24.8)	1485 (22.1)	10 957 (25.2)	0.08	<0.0001
Other	160 (0.3)	17 (0.3)	143 (0.3)	0.01	<0.0001
Vintage (median, IQR)	2.0 (0.4-5.4)	3.0 (0.8-6.7)	1.9 (0.4-5.2)	**0.25**	**0.19**
TT (minutes, median, IQR)	240 (210-240)	240 (230-240)	240 (210-240)	0.19	0.004
TT <3.5 hours	9745 (15.4)	706 (9.9)	9039 (16.1)	0.18	0.01
Dialysate K bath (median, IQR) (n = 27 776)	2.0 (2.0-2.0)	2.0 (2.0-2.0)	2.0 (2.0-2.0)	0.003	**0.27**
K gradient (potassium—dialysate K bath) (median, IQR) (n = 27 345)	2.7 (2.1-3.4)	3 (2.4-3.7)	2.7 (2-3.4)	**0.34**	**0.60**
Country/region					
Australia/New Zealand	2344 (4.7)	79 (1.2)	2265 (5.2)	**0.23**	<0.0001
Belgium	3268 (6.5)	637 (9.5)	2631 (6.1)	**0.13**	<0.0001
Canada	3687 (7.4)	460 (6.8)	3227 (7.4)	**0.02**	<0.0001
France	2473 (4.9)	1252 (18.6)	1221 (2.8)	**0.53**	<0.0001
Gulf Cooperation Council	1361 (2.7)	48 (0.7)	1313 (3.0)	**0.17**	<0.0001
Germany	3865 (7.7)	598 (8.9)	3267 (7.5)	**0.05**	<0.0001
Italy	3156 (6.3)	621 (9.2)	2535 (5.8)	**0.13**	<0.0001
Japan	10 790 (21.5)	1086 (16.1)	9704 (22.4)	**0.16**	<0.0001
Spain	4235 (8.5)	322 (4.8)	3913 (9.0)	**0.17**	<0.0001
Sweden	3830 (7.6)	1391 (20.6)	2439 (5.6)	**0.46**	<0.0001
Turkey	120 (0.24)	28 (0.42)	92 (0.21)	**0.04**	<0.0001
United Kingdom	3133 (6.2)	39 (0.60)	3094 (7.1)	**0.35**	<0.0001
United States of America	7885 (15.7)	174 (2.6)	7711 (17.8)	**0.52**	<0.0001

*Note.* SPS = sodium polystyrene sulfonate; N = number; % = percent; SD = standard deviation; GI = gastrointestinal; HTN = hypertension; PVD = peripheral vascular disease; CHF = congestive heart failure; CAD = coronary artery disease; DVT = deep vein thrombosis; PPI = proton pump inhibitor; ACEI = angiotensin-converting enzyme inhibitor; ARB = angiotensin receptor blocker; ASA = acetylsalicylic acid; mEq/L = milliequivalents per liter; AVF = arteriovenous fistula; AVG = arteriovenous graft; IQR = interquartile range; TT = treatment time; K = potassium.

a A standardized difference of 0.1 or greater would be considered a statistically significant difference. Significant differences are bolded.

## Results

### Characteristics of Study Cohort by SPS Use

From a total of 50 147 eligible patients during the study period, 6735 individuals (13.4%) received SPS. Baseline characteristics of the total SPS users and non-SPS users are listed in Table 1. Comparing SPS users with those without SPS, no difference was noted in the average age (64 years) or sex (60.6% male). Sodium polystyrene sulfonate users were less likely to have a history of diabetes and more likely to have had a parathyroidectomy. No differences were noted among other comorbidities or by medication use between the 2 groups. Pre-dialysis serum phosphate (5.5 vs 5.2 mg/dL) and potassium (5.2 vs 4.9 mEq/L) were higher in SPS users compared with non-SPS users. Sodium polystyrene sulfonate users were less likely to have a pre-dialysis potassium less than 6 mEq/L (83.9% vs 91.9%). When examining dialysis characteristics, SPS users were more likely to have an arteriovenous fistula (AVF), a longer dialysis vintage, longer treatment times, and a higher potassium gradient (defined as the serum potassium—the dialysate potassium bath). After weighting, significant differences persisted in mean serum potassium level, the percentage with a potassium value below 6 mEq/L, dialysis vintage, the dialysis potassium bath, and potassium gradient. Sodium polystyrene sulfonate use varied between countries with higher use in France (50.6%), Sweden (36.3%), Turkey (23.3%), Italy (19.7%), and Belgium (19.5%). Low use (<3%) was noted in Gulf Cooperation Council countries (3.5%), Australia/New Zealand (3.4%), the United Kingdom (1.2%) and the United States (0.2%). In Canada, SPS usage was 12.48%.

### Association of SPS With an Adverse GI Event

An adverse GI event occurred in 935 (1.9%) of the total cohort with 140 (2.1%) and 795 (1.9%) in SPS and no SPS users, respectively, with an absolute risk difference of 0.2%. Crude rates (per 1000 person-years) were as follows: GI hospitalization (SPS use: 8.4; no SPS use: 9.0), GI fatality (SPS use: 3.8, no SPS use: 2.9), and combined (SPS use: 11.8; no SPS use: 11.5). In weighted analyses, no difference in the risk for a GI event was observed among the SPS group compared with the non-SPS users (hazard ratio [HR] = 0.93; 95% confidence interval [CI] = 0.83-1.06) (Table 2). When examining GI events individually, no difference was observed with either fatal (248 events total; SPS use 0.1% vs 0.5% no SPS; HR = 0.87; 95% CI = 0.75-1.10) or GI hospitalization (723 events total; SPS use 1.5% vs 1.4%, HR = 1.10; 95% CI = 0.88-1.39). The median follow-up time was 1.5 years for total cohort. The median time to a GI hospitalization or a GI fatality was 9.3 and 10.7 months, respectively. These findings were consistent in additional models adjusting for dialysis vintage and the potassium gradient (Table 3). We examined both GI hospitalizations and GI fatality pre-2010 and 2010 onward to examine whether the FDA warning led to a change in outcomes. We found no change in either GI hospitalization (pre-2010: 1.48%, 2010 onward: 1.49%) or GI fatality (pre-2010: 0.63%, 2010 onward: 0.75%) in SPS users.

**Table 2.** Weighted Hazard Ratio for Gastrointestinal Outcomes (Composite, Fatal, and Non-Fatal Events) by Sodium Polystyrene Sulfonate (SPS) Use and Non-Use in an International Cohort of Patients on Hemodialysis in the Dialysis Outcomes and Practice Patterns Study (DOPPS).

Outcome	GI hospitalization^a^	Fatal GI event^b^	Composite GI event^b^
Weighted HR (95% CI)	0.87 (0.75, 1.00)	1.10 (0.88, 1.39)	0.93(0.83, 1.06)
No. of events/total cohort (%)	723/50 147 (1.4%)	248/49 155 (0.5%)	935/49 180 (1.9%)
SPS use: No. of events/total cohort (%)	100/6735 (1.5%)	45/6610 (0.1%)	140/6613 (2.1%)
No SPS use: No. of events/total cohort (%)	623/43 412 (1.4%)	203/42 545 (0.5%)	795/42 567 (1.9%)

*Note.* Weights were calculated using all variables listed in Table 1. GI = gastrointestinal; HR = hazard ratio; CI = confidence interval; N = number; % = percentage.

a Assessed in the total cohort of 50 147.

b Assessed in 49 155 individuals (992 excluded for missing cause of death).

**Table 3.** Additional Analyses Further Adjusting the Weighted Models by Dialysis Vintage and Potassium Gradient for Gastrointestinal Outcomes (Composite, Fatal, and Non-Fatal Events).

Outcome	GI hospitalization^a^	Fatal GI event^b^	Composite GI event^b^
Weighted HR (95% CI)	0.82 (0.68, 1.00)	1.29 (0.96, 1.73)	0.96 (0.82, 1.13)
No. of events/total cohort (%)	401/27 345 (1.5%)	154/26 984 (0.6%)	535/26 994 (2.0%)
SPS use: No. of events/total cohort (%)	56/3745 (1.5%)	30/3700 (0.8%)	84/3701 (2.3%)
No SPS use: No. of events/total cohort (%)	345/23 600 (1.5%)	124/23 284 (0.5%)	451/23 293 (1.9%)

*Note.* Weights were calculated using all variables listed in Table 1. Models were additionally adjusted for dialysis vintage and potassium gradient. GI = gastrointestinal; HR = hazard ratio; CI = confidence interval; SPS = sodium polystyrene sulfonate; N = number; % = percentage.

a Assessed in the total cohort of 50 147.

b Assessed in 49 155 individuals (992 excluded for missing cause of death).

## Discussion

In a large international cohort of individuals on long-term hemodialysis, we found no difference in the risk of an adverse GI event among individuals exposed to SPS use compared with those not exposed to SPS use. No difference was observed when we examined the severity of the GI event separately as fatal or non-fatal. Our findings were consistent in additional models accounting for slight imbalance post-weighting and when examining before and after SPS pre-mixing with sorbitol. Our results add to the growing literature on SPS use as a therapeutic agent for the management of hyperkalemia and suggest no detectable GI-related harm in the hemodialysis population.

Multiple reports link SPS use to mucosal injury of the GI tract.^8-23^ In a surgical uremic rat model, SPS enema given alone or with sorbitol or mannitol seemed to cause colon necrosis and high mortality rate, whereas 33% sorbitol without SPS did not.^34^ In 2013, a systematic review reported that in 17 cases out of the 58 described cases of GI injury, SPS did not contain sorbitol.^22^ Furthermore, the colon was the most common site of injury (76%, affecting also the small intestine and the upper GI tract), and transmural necrosis (62%) was the most common histopathologic lesion reported, while mortality was reported in 33% of these cases due to GI injury. In a population-based cohort study of 20 020 SPS users matched to non-users, SPS use was associated with a higher 30-day risk of GI injury requiring an emergency room visit or hospitalizations (0.2% with SPS vs 0.1% without SPS; HR = 1.94; 95% CI, 1.10-3.41).^20^ Furthermore, in a Swedish observational study (2006-2016), in patients with chronic kidney disease (CKD), the initiation of SPS was associated with a dose-dependent higher risk of severe GI complications (intestinal ischemia, thrombosis, or ulceration/perforation), as well as the initiation of GI-related medications, compared with non-SPS users.^25^

Our results contradict previous findings demonstrating no measurable risk with SPS use and are consistent with a recent French REIN registry study that reported no association with SPS or calcium polystyrene sulfonate (CPS) use and the risk of composite (fatal and non-fatal) GI injury.^25^ In models accounting for time-varying exposure, they found no association with SPS or CPS use and GI events of interest (occlusion, perforation, or thrombosis) or other GI events. Previous reports of GI injury with SPS were primarily in non-dialysis populations, or when individuals on dialysis were included, they were in small numbers. This does raise the possibility of a differential risk for those requiring dialysis versus the non-dialysis population. Other alternative explanations include differences in study design, handling of the drug exposure (accounting for dose and duration), and possible concurrent drug use that may limit/attenuate the risk (laxatives). The current study expands on these findings by including a larger cohort size with a higher number of events, by the incorporation of important dialysis-related factors such as vascular access, pre-dialysis serum potassium, dialysis time, treatment time, and potassium gradient; and by examining an international dialysis cohort. Our crude rates of GI injury are comparable to those of previous studies, further suggesting appropriate outcome capture.

Our study has limitations. Sodium polystyrene sulfonate exposure was determined by receipt of a prescription and information on adherence, length of use, and dosage were not available. The study exposure was a first SPS prescribed (new user) on periodic (updated every 4 months) medication review since dialysis initiation and our analysis was intention to treat. We did not have information on the route of SPS administration or whether it was co-administered with sorbitol. No pathological, imaging, or autopsy data were available, thus misclassification is possible. Our focus on GI composite events, as opposed to individual types of GI pathology, may partially mitigate this. Despite accounting for many variables in our weighted models, residual confounding may remain a possibility. Post-weighting, a few variables did remain imbalanced (related to potassium and dialysis vintage) that required further adjustment in our analytic models. Nevertheless, our study is the largest, international study capturing physician deemed cause of death. Our findings of no higher risk with SPS use were consistent when we examined different outcomes (fatal or any GI event).

## Conclusions

In a large international hemodialysis prospective cohort study, we found SPS use was not associated with a higher risk of a GI adverse event, and this was consistent in additional analyses. Further research to delineate the GI injury risk by dose-dependent SPS exposure and long-term use in the maintenance hemodialysis population is warranted.

## Supplemental Material

sj-docx-1-cjk-10.1177_20543581231172405 – Supplemental material for Adverse Gastrointestinal Events With Sodium Polystyrene Sulfonate Use in Patients on Maintenance Hemodialysis: An International Cohort Study Click here for additional data file. Supplemental material, sj-docx-1-cjk-10.1177_20543581231172405 for Adverse Gastrointestinal Events With Sodium Polystyrene Sulfonate Use in Patients on Maintenance Hemodialysis: An International Cohort Study by Ana Cecilia Farfan Ruiz, Ranjeeta Malick, Emily Rhodes, Edward Clark, Greg Hundemer, Angelo Karaboyas, Bruce Robinson, Roberto Pecoits and Manish M. Sood in Canadian Journal of Kidney Health and Disease
