# Supplementary material for: Adverse Gastrointestinal Events With Sodium Polystyrene Sulfonate Use in Patients on Maintenance Hemodialysis: An International Cohort Study
Source: Can J Kidney Health Dis. 2023 Jun 21;10:20543581231172405. doi: 10.1177/20543581231172405 (PMC10288443; doi:10.1177/20543581231172405)
Supplement: sj-docx-1-cjk-10.1177_20543581231172405 – Supplemental material for Adverse Gastrointestinal Events With Sodium Polystyrene Sulfonate Use in Patients on Maintenance Hemodialysis: An International Cohort Study [file sj-docx-1-cjk-10.1177_20543581231172405.docx]

**Supplement:**

Table 1: Cohort creation and exclusions

|  | Number excluded | Total cohort size |
| --- | --- | --- |
| Total DOPPS cohort |  | 51,996 |
| Vascular access data missing | 1,842 | 50,154 |
| End date missing | 7 | 50,147 |
| Cause of death unknown | 992 | 49,155 |
